# Homozygosity mapping in the Kazakh national dog breed Tazy

**DOI:** 10.1038/s41598-023-37990-5

**Published:** 2023-07-03

**Authors:** Anastassiya Perfilyeva, Kira Bespalova, Sergey Bespalov, Mamura Begmanova, Yelena Kuzovleva, Olga Vishnyakova, Inna Nazarenko, Gulnar Abylkassymova, Yuliya Perfilyeva, Konstantin Plakhov, Leyla Djansugurova, Bakhytzhan Bekmanov

**Affiliations:** 1 Department of Molecular Genetics, Institute of Genetics and Physiology, 050060 Almaty, Kazakhstan; 2 Department of Biology and Biotechnology, Al-Farabi Kazakh National University, 050040 Almaty, Kazakhstan; 3 Department of Theriology, Institute of Zoology, 050060 Almaty, Kazakhstan; 4 Republican Federation of Public Associations of Hunters and Hunting Societies “Kansonar”, 050008 Almaty, Kazakhstan; 5 Republican Federation of Public Associations of Hunters and Hunting Societies “Kansonar”, 020000 Astana, Kazakhstan; 6 Laboratory of Molecular Immunology and Immunobiotechnology, M.A. Aitkhozhin’s Institute of Molecular Biology and Biochemistry, 050012 Almaty, Kazakhstan; 7 Laboratory of Biocenology and Hunting Management, Institute of Zoology, 050060 Almaty, Kazakhstan

**Keywords:** Evolution, Genetics, Zoology

## Abstract

The Tazy is a breed of sighthound common in Kazakhstan. The identification of runs of homozygosity (ROH) is an informative approach to assessing the history and possible patterns of directional selection pressure. To our knowledge, the present study is the first to provide an overview of the ROH pattern in the Tazy dogs from a genome-wide perspective. The ROH of the Tazy was found to be mainly composed of shorter segments (1–2 Mb), accounting for approximately 67% of the total ROH. The estimated ROH-based inbreeding coefficients (F_ROH_) ranged from 0.028 to 0.058 with a mean of 0.057. Five genomic regions under positive selection were identified on chromosomes 18, 22, and 25. The regions on chromosomes 18 and 22 may be breed specific, while the region on chromosome 22 overlaps with regions of hunting traits in other hunting dog breeds. Among the 12 candidate genes located in these regions, the gene CAB39L may be a candidate that affects running speed and endurance of the Tazy dog. Eight genes could belong to an evolutionarily conserved complex as they were clustered in a large protein network with strong linkages. The results may enable effective interventions when incorporated into conservation planning and selection of the Tazy breed.

## Introduction

The Tazy is a well-known national sighthound dog in Kazakhstan. Previously it was shown that it is a genetically divergent ancient dog breed with a strong position in the phylogenetic tree and a high level of genetic diversity^1^. In recent years, it has become apparent that conventional breeding methods alone cannot lead to significant progress in this breed. Genetic research is required to improve the accuracy of the genomic evaluation of each dog and to make long-term genetic progress. In this regard, ROH analysis to estimate genome-wide inbreeding levels and selection signatures is a potential way to improve the efficiency and precision of conventional breeding.

ROH are defined as contiguous regions of the genome in which an individual is homozygous at different sites^2^. The number and length of ROH reflect individual and population history. Long, contiguous ROH segments (over 10 Mb) indicate recent inbreeding about five generations ago. Short ROH segments reflect distant or ancient inbreeding, as recombination allows for the breakdown of segments over time. Distant inbreeding can be classified as inbreeding events that occurred between 50 and 12.5 generations ago when ROH lengths range from 1 to 4 Mb, respectively^3–5^. The ability of ROH segments to provide information about genetic events in a population makes them a useful tool for studying the breeding process over time. In addition, ROH analysis provide useful information about genetic relatedness between individuals, helping to minimize inbreeding depression. It is known that the F_ROH_ is more accurate than inbreeding coefficient estimates from pedigree data in determining inbreeding effects^6^. Moreover, the F_ROH_ can be used to derive information about the degree of inbreeding when genealogical information is not available^6^. Finally, the extended blocks of homozygosity on a megabase scale appear to be best explained by selection, so studies using ROH may contribute to understanding the genetic basis of important traits or diseases^7^.

Since determining the extent of genomic inbreeding and patterns of selection pressure are fundamental to setting conservation and selection priorities, mapping of homozygosity has already been performed in Chinese indigenous dog breeds^8^, the Braque Français^9^, Bernese Mountain dogs^10^, Border Collie dogs^11^, Bullmastiff dogs^12^, German Shepherd^13^, and Livestock Guardian Dogs^14^. Using the ROH approach, variants involved in a number of morphological and behavioural traits have been identified in dog breeds^7^.

As far as we know, the ROH patterns in the Tazy have not been studied yet. The aim of this study was to analyze the genetic history and genome-wide signals of positive selection, as well as to evaluate genomic inbreeding in the Tazy dogs using a ROH approach.

## Results

### Characteristic of ROH

**Figure 1:**
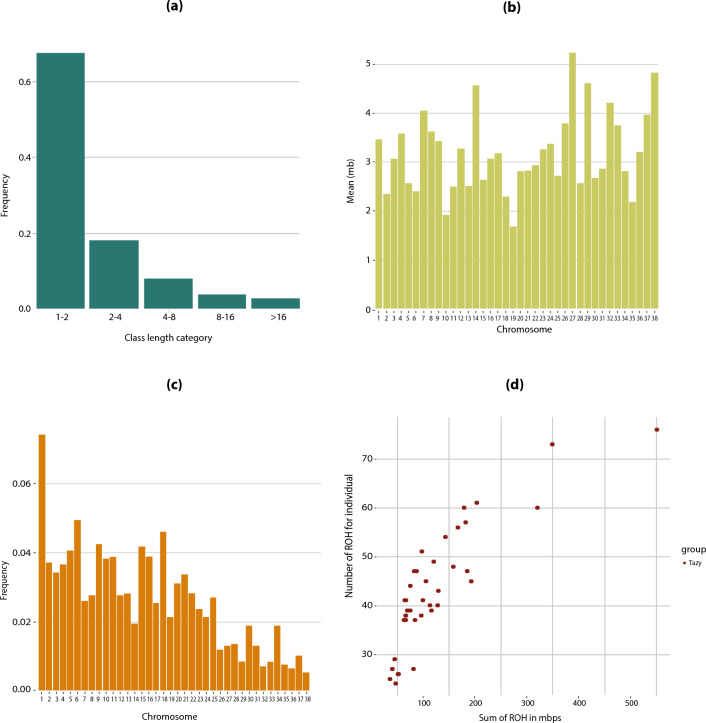
ROH characteristics: (**a**) Frequencies of ROH for each length class. (**b**) Mean length (Mb) of ROH for each chromosome. (**c**) Frequencies of ROH per chromosomes, (**d**) Relationship between ROH number per dog and total length of the genome covered by ROH. Each point stands for one dog.

A total of 1699 ROH were identified in all 39 Tazy dogs (Supplementary Table S1). The ROH were predominantly short. In total, there were 1143 ROH of 1–2 Mb in length (67%), 306 ROH of 2–4 Mb (18%), 136 ROH of 4–8 Mb (8%), 67 ROH of 8–16 Mb (4%), and 47 ROH > 16 Mb (3%) (Fig. 1a). Short ROH of 1–2 Mb in length covered 1.62% of the genome, the largest proportion compared to the other groups (see Table 1). Overall, the proportion of the genome covered by ROH was 5.37%.

**Table 1:** Characteristics of ROH and F_ROH_ of the Tazy for different length classes.

Length class	Average length of ROH (Mb)	Average number of ROH per animal	Genome coverage (%)	Mean F_ROH_	Min F_ROH_	Max F_ROH_
1–2	1.33	29.31	1.62	0.058 ± 0.045	0.016	0.249
2–4	2.68	7.85	0.88	0.041 ± 0.043	0.005	0.228
4–8	5.63	3.49	0.82	0.033 ± 0.041	0.002	0.211
8–16	11.02	1.72	0.79	0.035 ± 0.042	0.004	0.198
> 16	25.13	1.21	1.26	0.028 ± 0.032	0.007	0.137
Total			5.37			

ROH were found on all chromosomes. A graphical representation of the mean length and frequency of ROH for each chromosome is shown in Fig. 1b and c, respectively. The longest ROH were observed on chromosome 27 (5.16 Mb) and the shortest on chromosome 19 (1.66 Mb). The highest number of ROH was detected on chromosome 1 (126 ROH) and the lowest on chromosome 38 (9 ROH). The number of ROH per dog ranged from 24 to 76, with an average number of ROH for the sample of 43.56 ± 12.31. The relationship between ROH number per dog and the total length of the genome covered by them is presented in Fig. 1d. Most individuals clustered near the coordinate origin, which could be due to the frequency of the shorter ROH. The six longest ROH (> 35 Mb) were found in three dogs: T17, T86, and T90 (see Supplementary Table S1).

### The distribution of eROHi (extreme runs of homozigosity islands)

**Figure 2:**
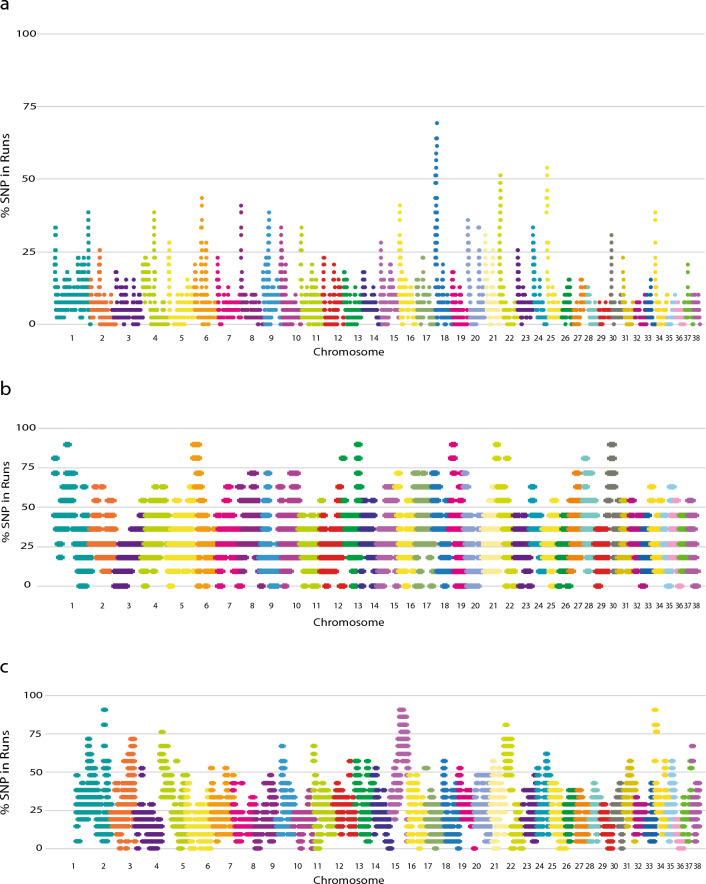
Manhattan plot of the distribution of ROH in the genome of the Tazy (**a**), Afghan Hound (**b**) and Saluki (**c**).

The genomic distribution of overlapping ROH of the Tazy was inconsistent in length and position on chromosomes. Figure 2a shows the SNP occurrences in ROH across the genome. The highest chromosomal peaks were found on chromosomes 18, 22, and 25.

A total of five eROHi on these chromosomes were identified in at least 20 samples (see Table 2). The strongest pattern was observed on chromosome 18, where an overlapping ROH region was present in 27 Tazy dogs. To determine if the identified eROHi are breed specific for the Tazy, we found overlapping ROH regions for the closest relatives of the Tazy breed, the Saluki and the Afghan Hound. The genomic regions with the highest frequency of ROH were found on chromosomes 1, 6, 13, 19, 22, and 30 in the Afghan Hound and on chromosomes 1, 2, 4, 6 ,15, 22, and 34 in the Saluki (Fig. 2b and c, respectively). The location and size of the identified eROHi in these two breeds can be found in Supplementary Table S2.

**Table 2:** Characteristics of eROHi of the Tazy.

eROHi*	Chr	nSNP^#^	from	to	Size (kb)	nDog^$^	Genes
1	18	15	913,868	1,221,882	308,014	20	LOC111090815, LOC111090792
2	18	17	1,272,707	1,583,327	310,620	21	VWC2, ZPBP, LOC119864151, SPATA48
3	18	57	3,319,077	4,393,071	1073,994	27	LOC606902, LOC100687611,
							LOC119864094,
							LOC119864356, LOC111090858
4	22	69	2,029,245	2,904,155	874,910	20	KPNA3, EBPL, LOC102151128, RCBTB1, SETDB2, LOC119865111, CAB39L,
							CDADC1, LOC119865167, LOC102152970, MLNR,LOC119865166,FNDC3A,LOC111091797,
							LOC119865168, LOC111091799,
							LOC102153123, LOC119865169,
							LOC119865170, CYSLTR2
5	25	17	863,981	1,230,408	366,427	21	LOC102153908, LOC111092399, LOC119865890, LOC106557760, LOC119865889, LOC100683497, LOC100683578, LOC02153976

*Extreme Runs of Homozygosity islands.^#^Number of SNPs within each eROHi. ^$^Number of individuals that occurred in each eROHi.

### The functional relevance of eROHi of the Tazy

A total of 39 genes were present in the five eROHi of the Tazy (see Table 2), including 12 candidate genes with known functional significance (ZPBP, SPATA48, VWC2, KPNA3, EBPL, RCBTB1, SETDB2, CAB39L, CDADC1, MLNR, FNDC3A, and CYSLTR2). The annotation of these genes and the functional characteristics according to the categories GO and KEGG are shown in Supplementary Tables S3 and S4, respectively. Overall, they were found to be involved in 39 GO terms (14 biological processes, 15 cellular components, and 10 molecular functions) and 9 KEGG pathways. In the BP category, candidate genes were most enriched in the G protein-coupled receptor signaling pathway (GO:0,007,186), whereas in the CC category, candidate genes were most enriched in the cytoplasm (GO:0,005,737), plasma membrane (GO:0,005,886), and integral component of the membrane (GO:0,016,021). The most enriched molecular functions and KEGG pathways were associated with protein binding (GO:0,005,515), G protein-coupled receptor activity (GO:0,004,930), and interaction between neuroactive ligands and receptors (cfa04080), respectively.

**Figure 3:**
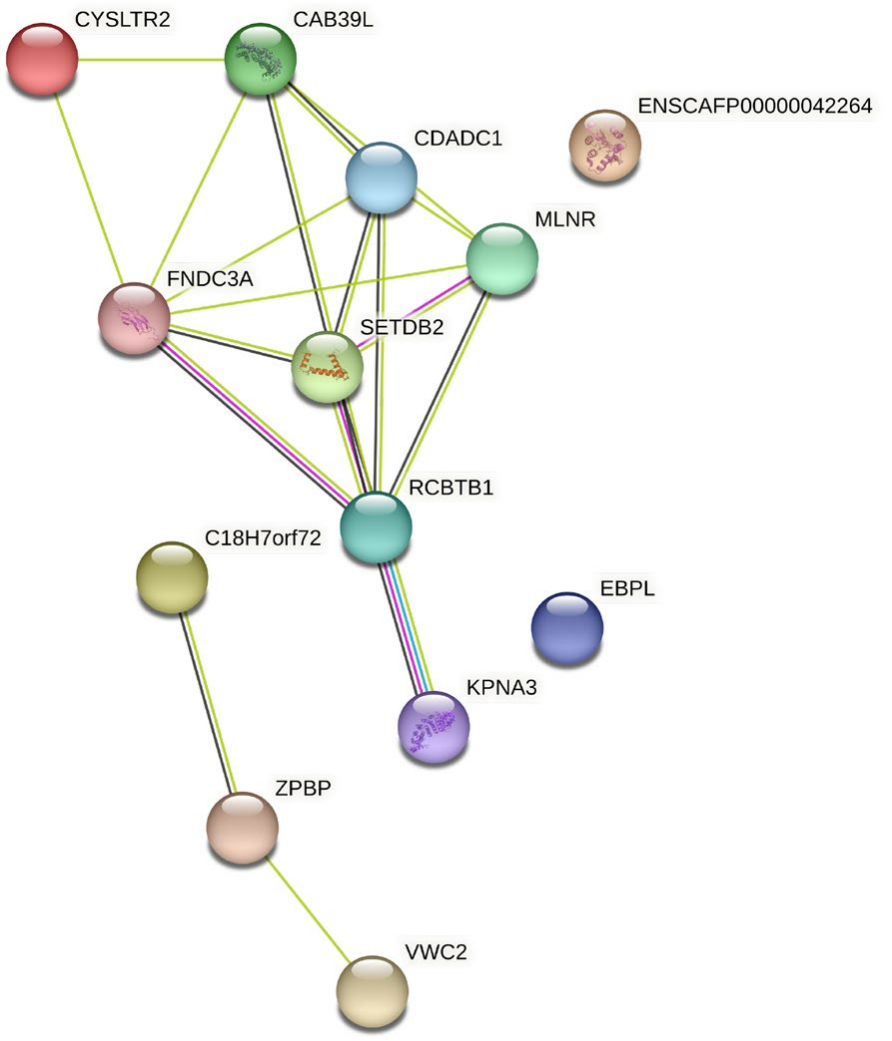
PPI network for genes mapped in eROHi.

Interestingly, eight genes, namely KPNA3, RCBTB1, SETDB2, CAB39L, CDADC1, MLNR, FNDC3A, and CYSLTR2 from the annotated genes were clustered in a large PPI network (Fig. 3) and showed significantly more interactions than expected (PPI enrichment p-value < 1.0e-16).

A total of 175 eROHi_SNPs were mapped in five eROHi, of which 58 eROHi_SNPs were found in candidate genes ZPBP, SPATA48, VWC2, KPNA3, EBPL, RCBTB1, SETDB2, CAB39L, CDADC1, MLNR, FNDC3A, and CYSLTR2. Most SNPs were mapped in intronic (49%) and intergenic positions (41%). The only SNP missense position (rs23023309) was found in the CAB39L gene. The detailed Ensembl VEP annotation of the eROHi_SNPs is shown in Supplementary Table S5.

### ROH-based inbreeding

**Figure 4:**
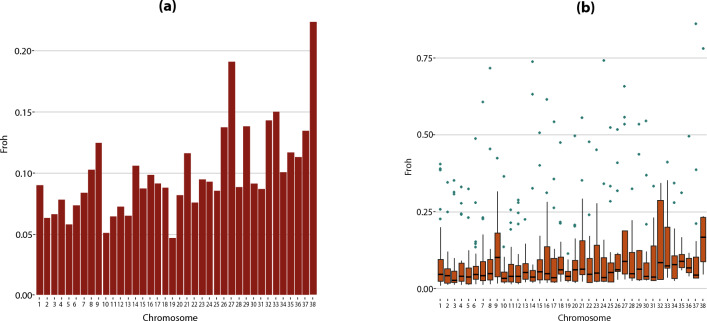
F_ROH_ of the Tazy dogs. (**a**) Average F_ROH_ per chromosome. (**b**) The distribution of inbreeding by chromosome, calculated as the proportion of the chromosome in ROH over the length of the chromosome covered by involved SNPs. The black dots are maximum values of F_ROH_.

F_ROH_ was estimated for each chromosome and dog. The highest mean F_ROH_ values were determined for chromosomes 27, 33 and 38 (Fig. 4a). The mean value of F_ROH_ per dog was 0.057 ± 0.045 with a range from 0.017 to 0.250 (Supplementary Table S6). Three dogs had high F_ROH_ values > 0.1 (dogs T120, T86, and T90). Two of them showed extremely high F_ROH_ values per chromosome: F_ROH_ was > 0.7 for chromosomes 8, 14, 24, 37 in dog T86 and for chromosome 38 in dog T120 (Fig. 4b). The highest F_ROH_ value was observed for ROH 1–2 Mb in length (0.058) and ranged from 0.016 to 0.249 (Table 1).

## Discussion

In this study, for the first time, we presented an overview of the ROH patterns of the Kazakh national dog breed Tazy from a genome-wide perspective. According to the obtained results, there was strong evidence of distant inbreeding in this breed about 50 generations ago, as most ROH fell into the short (1–2 Mb) category, which allowed ROH decay by recombination over a long period of time^5^. Since the generation length of the Tazy is 1.7–3.1 years, it is likely that the genetic diversity of the Tazy was influenced by the social and climatic disasters in Kazakhstan in 1868–1938. The Russian-Kokand War (1850–1868) took place throughout southern Kazakhstan, which may have been the place of origin of the Tazy ^15^ and was probably the main area of Tazy dogs during that period. World War I (1914–1918), in which the indigenous population of Kazakhstan was partially mobilized, the mass starvation of livestock due to ice in Kazakhstan in the late 19th and early twentieth centuries, and the collectivization of a large number of farms in the 1930s may have indirectly affected the Tazy population by leading to a decline in population and economy.

The mean ROH-based inbreeding coefficient per dog (0.057 ± 0.045), which ranges from 0.028 to 0.058 for the different length classes, indicates a low level of inbreeding in the Tazy dog. It is like the inbreeding coefficient estimated from homozygosity runs for the Jack Russell terrier (0.061)^12^ and lower than for the Braque Français (0.112 ± 0.023)^9^, German Shepherd (F_ROH_ for lengths > 1 MB–0.119)^13^, and the Bulldog (0.151)^12^, but did not reach the level of F_ROH_ for wild dogs. For example, the F_ROH_ for African wild dogs (*Lycaon pictus*) is much lower (0.0045 ± 0.0012)^5^. These results, combined with the previously shown high diversity of the Tazy breed ^1^, suggest that the current genetic status of the Tazy dog population is comparable to that of diverse breeds such as the Jack Russell terrier^16^. Therefore, the long-term survival of this breed is unlikely to be affected by deleterious genetic factors associated with inbreeding depression. However, three of the 39 Tazy dogs studied were highly inbred, indicating a practice of consanguinity that should be considered in future breeding strategies.

In addition to demographic history and inbreeding evaluation, ROH analysis is an effective approach to determine the direction of genetic selection^6,17^. Homozygous sequences are probably not randomly distributed across genomes. The ROH patterns shared by a large proportion of individuals in a population can be used to identify genomic regions that contain traces of genetic selection. Our analysis focused on the genomic regions with the highest frequency of ROH, that were presented in more than 50% of the Tazy dogs. The five strongest signals were found on chromosomes 18, 22, and 25. Regions of chromosomes 18 and 22 include twelve candidate genes with known functional significance: ZPBP, SPATA48, VWC2, KPNA3, EBPL, RCBTB1, SETDB2, CAB39L, CDADC1, MLNR, FNDC3A, and CYSLTR2.

The most notable genes from this list are ZPBP, SPATA48, FNDC3A, and CYSLTR2, which play important roles in spermatogenesis and fertilization. Genes regulating spermatogenesis represent a category of commonly enriched genes in many mammalian species^18^. Further analysis showed that a ~ 500 kb region on chromosome 22 containing the FNDC3A and CYSLTR2 genes is under selection in dog breeds with a hunting background (Beagle, Border terrier, English Bulldog, Gordon Setter, Irish Wolfhound, Newfoundland, Rottweiler, Weimaraner)^7^. Akkad et al. identified a longer region (~ 1,0 Mб) on chromosome 22 while comparing pointing and herding dogs^19^. This region contains the candidate genes CDADC1, MLNR, RCBTB1 и SETDB2, in addition to the FNDC3A and CYSLTR2 mentioned above, which agrees well with our study. Akkad et al. showed that only dogs of the pointing dog breeds (English Setter, German Longhaired Pointing dog, Gordon Setter, Irish Setter, Great Munsterlander, and Weimaraner) were homozygous for this region, which was significantly different from the herding dog breeds (Berger des Pyrenées, Giant Schnauzer, Kuvasz, and Schapendoes). The authors suggested that the region of chromosome 22 is a prerequisite for pointing. In our study, the region of chromosome 22 (~ 900 kb) also shows strong evidence for positive selection in the Tazy breed. The candidate genes KPNA3, EBPL, RCBTB1, SETDB2, CAB39L, CDADC1, MLNR, FNDC3A, and CYSLTR2 in this region were clustered in a large PPI network with strong linkages. But hunting with a Tazy dog differs from hunting with a pointing dog. Pointing dogs are trained to find a prey animal and indicate its location so the hunter can approach and flush it, while hunting with the Tazy is called sonar. In sonar, the Tazy dog searches for the prey animal, catches and kills it or holds it until the hunter-rider arrives. It is possible that the region of chromosome 22 is important for the hunting characteristics of both a sighthound and a pointing dog, such as sensory perception, search field activity, and the ability to think at the pace of one's movement. This hypothesis agrees well with the fact that the Afghan Hound and Saluki had similar regions of chromosome 22 under selection in our analysis (~ 200 kb and ~ 2.6 Mb, respectively).

Interestingly, only in two Chinese hunting sighthounds (Liangshan, Qingchuan) was no evidence of selection found in any of the regions of chromosome 22 that we have identified, possibly due to the evolution of their hunting abilities during adaptation to high altitude^8^. In addition, the Shaanxi Xi dogs had other SNP outliers despite their phenotypic similarity to the Tazy dogs ^8^. The newly available genotypic data from these dogs will enable future phylogenetic analyzes to understand their relationship with the Tazy dogs and the differences in their selection.

Functional annotation of candidate genes identified in eROHi of chromosome 22 confirmed the enrichment of terms that may influence traits of interest to hunters. The most enriched molecular function was the G protein-coupled receptor protein signaling pathway, which is primarily attributed to olfactory signal transduction^20,21^. Among the KEGG terms, a signaling pathway related to the processing of environmental information, such as neuroactive ligand-receptor interaction, has been identified. The gene CAB39L has been linked to the positive regulation of the AMP-activated protein kinase (AMPK) pathway, which in humans maintains energy homeostasis during exercise^22^. Since the AMPK activator AICAR (5-amino-1-β-D-ribofuranosyl-imidazole-4-carboxamide) increases running endurance in mice^23^, the CAB39L gene may be critical for the outstanding running ability of Tazy dogs. It is known that the Tazy dog can accelerate up to 80 km per hour and track prey for a very long period. Perhaps the CAB39L gene is the strongest candidate in our study, as the only SNP missense position (rs23023309) was found in this gene.

While the functional significance of some candidate genes was clear, the involvement of others was unexpected. Among the genes with strong selection signal was the KPNA3 gene. The gene is associated with nuclear protein import and therefore plays a role in *Salmonella* infection processes, as the bacterial pathogen has been shown to manipulate host cell immune responses by interfering with the nuclear transport mechanism^24^. The possible significance of this mechanism for the positive selection of the Tazy is unclear, given the historically privileged position of the Tazy and the carefully considered nature of their diet. However, most of the dogs with this selective signal were from the northern region of Kazakhstan, which historically has the highest incidence of salmonellosis. In addition, in the north of Kazakhstan in the nineteenth century there was a loss of norms and traditions related to the keeping of Tazy dogs, when their diet included even food scraps.

Unfortunately, the functional significance of the remaining genes on chromosomes 18 (chr18:913,868–1,221,882 and chr18:3,319,077–4,393,071) and 25 (chr25:863,981–1,230,408) is still unknown. We have also not found orthologs for these genes in humans, rabbits, and rats. There is a possibility that the regions on chromosomes 18 and 25 are breed specific for the Tazy, as the candidate regions of these chromosomes have not overlapped with previous studies in other breeds. In the Braque Français, a French hunting dog breed, such genomic regions were identified on chromosomes 9, 15, 30, and 36, in addition to the region on chromosome 22 that appears to play a role in the phenotypes of most hunting dogs ^9^. In Bernese Mountain dogs, eROHi were identified on chromosomes 1, 2, 6, and 14^10^. In Border Collie, the highest frequency of SNPs in ROH was found on chromosomes 2, 5, 14, 24 and 26^11^. Moreover, the closest relatives of the Tazy breed, the Saluki and the Afghan Hound, do not have selective signals on chromosomes 18 and 22, as our analysis shows. Further study of these regions will be of great importance to uncover the genetic basis of differences between dog breeds.

A limitation of the study is the relatively small sample size due to the low number of purebred Tazy dogs with the highest expert scores in our country. Nevertheless, it provided important initial information for the conservation and breeding of this unique breed. In addition, only the eROHi approach was used in this study to identify selection signals. A future study should focus on comparing the obtained results with the results of other complementary and effective approaches, such as the integrated haplotype score (iHS)^25^ and the number of segregating sites by length (nSL) ^26^, to find the most reliable selection signal in the Tazy breed. Moreover, the X chromosome has a high gene density and a lower recombination rate^27^ and may therefore be a good target for detecting selection signatures^28^. Further enlargement of samples and analysis of selection patterns on the X chromosome certainly leave much room for a better understanding of selection processes.

## Conclusion

In the current study, we investigated the homozygosity of 39 Tazy dogs using a high-density genotyping array consisting of > 170,000 SNPs. We found evidence of a historical bottleneck in the Tazy population about 50 generations ago. The degree of genomic inbreeding showed that the Tazy breed has high genetic variability. Deciphering the selection signatures led to the identification of five strong regions on chromosomes 18, 22, and 25. The regions on chromosomes 18 and 22 may be breed specific. The region on chromosome 22 overlaps with the regions of hunting traits of other hunting dog breeds, including the closely related Afghan Hound and Saluki. Among the 12 candidate genes that showed the strongest selection signals, the CAB39L gene may be a candidate that affects the running speed and endurance of the Tazy dog. This study provides new insights into the history and selection of the Tazy breed.

## Material and methods

### SNP genotyping data

In this study, we used SNP genotype data from 39 Tazy dogs (25 females and 14 males) obtained with an Illumina Infinium CanineHD Genotyping BeadChip (Illumina Inc. San Diego, CA) from our previous study^1^. All procedures with animals in this study conformed to the guidelines of ARRIVE, were approved by the Ethics Committee of the Institute of Human and Animal Physiology, Almaty, Kazakhstan (number 3, September 15, 2020), and were performed in accordance with the relevant policies and regulations of our institution. All owners gave their written consent to use samples from their dogs for genetic studies.

In addition, publicly available SNP array data from 11 Afghan Hound dogs (3 females and 8 males) and 21 Saluki dogs (5 females, 2 males and 14 with missing sex) from the Dryad repository (datadryad.org, doi:10.5061/dryad.v9t5h;doi:10.5061%2Fdryad.pm7mt) were used in the study.

### Quality control and ROH analysis

Quality control and ROH analyses were performed using PLINK v1.9^29^. In the input report, 172,115 SNPs of the 39 Tazy dogs, 166,171 SNPs of the 11 Afghan Hound dogs, and 198,983 SNPs of the 21 Saluki dogs were filtered using the following steps (PLINK commands in brackets): only autosomal SNPs were retained (–not-chr X,Y,MT), the proportion of identity by descent (IBD) between two individuals was set to more than 0.4 (–genome; PI_HAT > 0.4), the individual call rate was set to 0.90 (–mind 0.10; did not apply to Saluki because of the many missing genotype data), and the minimum SNP call rate was set to 0.95 (–geno 0.05). Neither minor allele frequency pruning (–maf), no Hardy–Weinberg equilibrium test (–hwe), or LD pruning was performed^30^. The number of SNPs retained for calculations after the filtering process was 164,310 SNPs of the Tazy, 160,303 SNPs of the Afghan Hound, and 133,013 SNPs of the Saluki.

ROH segments were determined using PLINK v.1.9 with an overlapping window approach (–homozyg). A 50-SNP long sliding window was used to scan the genome (–homozyg-window-snp). All ROH detections were performed with less than a 1000 kb gap (–homozyg-gap) between adjacent ROH and a density of SNP coverage within the ROH of no more than 50 kb/SNP (–homozyg-density). The proportion of homozygous overlapping windows was 0.05 (–homozyg-window-threshold). The minimum number of SNPs forming a ROH was calculated using the L parameter (–homozyg-snp) following Lencz et al.^31^ and Purfield et al.^32^. The minimum length of an ROH was set at 1 MB to exclude short ROH (–homozyg-kb), as many of them might be due to inheritance of common allozygous haplotypes^33^. One heterozygous SNP was allowed (–homozyg-het) and one SNP could be missing (–homozyg-window-missing)^34^.

ROH were divided into five different categories according to their length: 1–2 Mb, 2–4 Mb, 4–8 Mb, 8–16 Mb, and > 16 Mb. Genome coverage by each ROH class was calculated by multiplying the average number of ROH per animal by the average ROH length, then dividing by the total ROS_Cfam_1.0 genome size (2396.86 MB), and finally multiplying by 100 to obtain the percentage value^35^. Graphical representations of ROH were obtained using the R package DetectRUNs^36^.

The eROHi were identified by selecting the SNPs most abundant in ROH ^37^, i.e., those that were present in at least 50% of samples of the Tazy. For the Afghan Hound and the Saluki, the threshold was 0.9 and 0.7, respectively, due to a limited sample.

The NCBI Map Viewer of the ROS _Cfam_1.0 (GCF_014441545.1) genome was used to identify genes in the eROHi (https://www.ncbi.nlm.nih.gov/genome/gdv?org=canis-lupus-familiaris&group=caniformia). Gene ontology (GO) analysis including the cellular component (CC), molecular function (MF), and biological process (BP)^38^ and Kyoto Encyclopedia of Genes and Genomes (KEGG) pathway analysis^39^ were performed for these genes using the R/Bioconductor package BioMart^40^ and the Database for Annotation, Visualization and Integrated Discovery (DAVID) (DAVID Bioinformatics Resources (ncifcrf.gov)^41^. GOLF: Gene and Ortholog Location Finder (https://rgd.mcw.edu/rgdweb/ortholog/start.html) were used to search for orthologs. The STRING database (version 11.5; https://string-db.org) was used to predict protein–protein interaction relationships (PPI) between annotated genes^42^.

The SNPs in the eROHi are referred as eROHi_SNPs. The eROHi_SNPs were annotated on the ROS _Cfam_1.0 (GCF_014441545.1) genome using the Variant Effect Predictor (VEP) of the Ensembl genome browser (http://asia.ensembl.org/info/docs/tools/vep)43.

A F_ROH_ was estimated for each dog and chromosome as the sum of all ROH divided by genome (or chromosome) length using the method described by McQuillan et al.^44^.

### The evaluation of the generation length of Tazy dog

Two methods were used to determine the generation length of the Tazy dog^45^. In the first method, pedigree analysis was performed, and the lifespan of several generations was divided by the number of generations. The analysis of 15 pedigrees showed that the average length of a generation of the Tazy dog was 3.11 ± 0.65 years. In the second method, the length of a generation was calculated as the sum of the average age at mating and the duration of gestation. The age at first estrus in the Tazy is nine months, as is the length of time between estruses. The duration of gestation is two months. Thus, the generation length of Tazy dog is 1.7 years.

## Supplementary Information

Supplementary Tables.

## Data Availability

The genotype data of the Tazy analysed during this study are available in the previously published article^1^. The genotype data of the Afghan Hound and the Saluki are available in the Dryad repository (datadryad.org, doi:10.5061/dryad.v9t5h;doi:10.5061%2Fdryad.pm7mt).
